# Patients on subcutaneous allergen immunotherapy are at risk of intramuscular injections

**DOI:** 10.1186/1710-1492-10-22

**Published:** 2014-05-07

**Authors:** Laura Kim, Immaculate Nevis, Ryan Potts, Clark Eeuwes, Arunmozhi Dominic, Harold L Kim

**Affiliations:** 1 Department of Anatomy and Cell Biology, McGill University, Montreal, QC, Canada; 2 Michael D DeGroote School of Medicine, McMaster University, Hamilton, ON, Canada; 3 Department of Biology, University of Waterloo, Waterloo, ON, Canada; 4 Schulich School of Medicine & Dentistry, Western University, London, ON, Canada

**Keywords:** Allergen-specific immunotherapy, Subcutaneous immunotherapy, Ultrasound, Skin-to-muscle depth, Needle length, Allergy syringe, Injections

## Abstract

**Background:**

Allergen-specific subcutaneous immunotherapy is an effective treatment for certain allergic disorders. Ideally, it should be administered into the subcutaneous space in the mid-posterolateral upper arm. Injections are commonly given using a standard allergy syringe with a needle length of 13 mm. Therefore, there is a risk of intramuscular administration if patients have a skin-to-muscle depth <13 mm, which may increase the risk of anaphylaxis. The objective of this study was to determine whether the needle length of a standard allergy syringe is appropriate for patients receiving subcutaneous immunotherapy.

**Methods:**

Ultrasounds of the left posterolateral arm were performed to measure skin-to-muscle depth in 200 adults receiving subcutaneous immunotherapy. The proportion of patients with a skin-to-muscle depth >13 mm vs. ≤13 mm was assessed and baseline characteristics of the two groups were compared. The proportion of patients with skin-to-muscle depths > 4 mm, 6 mm, 8 mm and 10 mm were also calculated. Multivariable logistic regression was performed to identify predictors of skin-to-muscle depth.

**Results:**

Of the 200 patients included in the study, 80% had a skin-to-muscle depth ≤13 mm; the majority (91%) had a skin-to-muscle depth >4 mm. Body mass index was found to be a significant predictor of skin-to-muscle-depth.

**Conclusions:**

Most patients receiving subcutaneous immunotherapy have a skin-to-muscle depth less than the needle length of a standard allergy syringe (13 mm). These patients are at risk of receiving injections intramuscularly, which may increase the risk of anaphylaxis. Using a syringe with a needle length of 4 mm given at a 45° angle to the skin may decrease this risk.

## Background

Allergen-specific subcutaneous immunotherapy (SCIT) is an effective treatment for allergic disorders such as allergic rhinitis/conjunctivitis and asthma [1-5]. It is recommended for patients with these conditions who have not improved clinically following avoidance of their identified allergens and the use of other guideline-recommended medical therapies. SCIT is typically given for 3 to 5 years [6], and successful treatment can lead to the resolution of allergic symptoms, even after the completion of therapy [6,7]. However, there is a potential risk of severe systemic reactions with SCIT, including anaphylaxis. The estimated risk of systemic reactions is 10 per 10,000 injection visits [8-15]. In one study, 4% of patients receiving SCIT had systemic reactions requiring epinephrine over one year [15]. Although rare, even fatal reactions to SCIT have been reported in large-scale surveys conducted by the American Academy of Allergy, Asthma and Immunology (AAAAI). These surveillance studies identified 41 deaths from SCIT between 1990 and 2001 [12,14,16].

SCIT is given in physicians’ offices using widely available allergy syringes. A standard syringe used in Canada has a needle length of 13 mm (*BD Safety Glide™ Allergy*). The AAAAI recommends that the needle be injected subcutaneously in the lateral or posterior portion of the arm since these areas tend to have a greater amount of subcutaneous tissue than adjacent areas [6]. It has been postulated that subcutaneous administration results in the formation of a reservoir of allergen extract that is absorbed slowly. Intramuscular injections should be avoided, as they may be associated with more rapid absorption of the extract, which could lead to an increased risk of systemic reactions [6]. In patients with a subcutaneous tissue depth less than the needle length of the allergy syringe, there is a possibility of inadvertent intramuscular injection of the allergen extract during SCIT. This study was performed to determine the subcutaneous tissue depth, that we will refer to as skin-to-muscle depth (STMD), in patients who receive SCIT in order to establish whether the needle length of a standard allergy syringe is appropriate.

## Methods

Participants who were receiving SCIT for aeroallergens at an allergy and immunology clinic in Kitchener, Ontario, Canada from April 2011 to December 2012 were included in this study. Subjects were receiving the SCIT in one or both arms depending on whether one or two sets of serum were required for appropriate treatment. SCIT was injected into the posterolateral upper arm(s) by a clinic physician or registered nurse using *BD Safety Glide™ Allergy* needles. Injections were typically given at approximately a 45° angle to the skin. All participants between 18 and 65 years of age had an ultrasound of the left posterolateral upper arm (injection site). After the ultrasound was completed, the injections were not given deeper than the STMD. Participants were excluded if they were younger than 18 years or over 65 years of age.

All ultrasounds were completed by a single physician using a Sonosite Titan® ultrasound machine. The depth from the outer skin to the inner aspect of the muscle fascia, or STMD, was measured on patients’ left mid-posterolateral upper arm. Based on this measurement, patients were divided into two groups: those with a STMD >13 mm and those with a STMD ≤13 mm. All patients provided informed consent and the study was approved by the Research Ethics Board at McMaster University.

Baseline characteristics of the two STMD groups were compared using two sample *t* tests or the Mann–Whitney *U* test for continuous variables depending on the distribution. Categorical variables were compared using the χ [2] or Fischer’s exact test. The proportions of patients with STMD greater than 4 mm, 6 mm, 8 mm and 10 mm were also calculated. Predictors such as age, sex, race, and body mass index (BMI) were forced into the multivariable logistic regression model. The model was selected using a forward stepwise process for entry at p ≤ 0.05 and removal at p ≥ 0.10 significance. All tests were two-sided with a level of statistical significance set at p ≤ 0.05. All statistical analyses were performed using Predictive Analytics SoftWare (SPSS V21.0).

## Results

**Figure 1:**
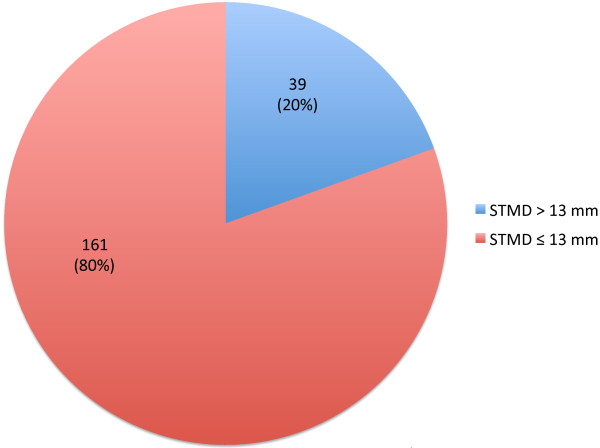
Proportion of SCIT patients with STMD >13 mm or ≤13 mm.

A total of 200 adult patients receiving aeroallergen SCIT were included in this study (see Table 1 for baseline characteristics of the study cohort). The mean age of all participants included in the study was 40.4 ± 13.8 years; 20% (39/200) of subjects had a STMD >13 mm and 80% (161/200) had a STMD ≤13 mm (see Figure 1). Gender and BMI were significantly different between these two STMD groups (Table 1). As expected, mean BMI was significantly higher in patients with a STMD >13 mm (33.1 kg/m [2]) compared to those with a STMD ≤13 mm (26.5 kg/m [2]) (p = 0.004). Interestingly, all participants with a STMD >13 mm were female. There were no significant differences in age or race between the two groups.

**Table 1:** Baseline characteristics of the entire study cohort and according to STMD ≤ 13 or > 13 mm

**Characteristics**	**All patients N = 200**	**Patients with STMD ≤ 13 mmn = 161**	**Patients with STMD > 13 mmn = 39**	P value*
Age, mean ± SD	40.4 ± 13.8	39.5 ± 13.7	44.3 ± 13.5	0.06
Number of males, (%)	80 (40)	80 (49.7)	0 (0)	0.0001
BMI, mean ± SD	27.8 ± 12.6	26.5 ± 13.4	33.1 ± 6.1	0.004
Number of Caucasians (%)	185 (93)	148 (92)	37 (95)	0.74

*calculated by student *t*-test or Fischer’s exact test.

STMD: skin-to-muscle depth; BMI: body mass index; SD: standard deviation.

The proportion of patients with STMD greater than 4 mm, 6 mm, 8 mm and 10 mm are shown in Table 2. This analysis revealed that the majority of patients had a STMD > 4 mm (91%). In a multivariable logistic regression analysis, BMI was found to be the only significant predictor of STMD (p = 0.015).

**Table 2:** Proportion of patients at different skin-to-muscle depths

**STMD**	**Number of patients (n)**	**Proportion of patients (%)**
> 4 mm	182	91
> 6 mm	149	75
> 8 mm	110	55
> 10 mm	74	37

## Discussion

SCIT is an effective and commonly used treatment for allergic conditions such as allergic rhinitis and asthma. Current guidelines recommend that injections be administered subcutaneously in the mid-posterolateral upper arm to allow for slow absorption of the allergen extract, thereby decreasing the risk of systemic reactions to SCIT. Our study using routine ultrasound in 200 adult patients receiving SCIT found that 80% of these patients had a STMD in the left posterolateral upper arm of less than 13 mm. The standard syringe used for SCIT has a needle length of 13 mm (*BD Safety Glide™ Allergy)*. We assumed the “worst case scenario” of the SCIT being given to maximal depth of the 13 mm needle as the techniques for giving injections will vary from one health practitioner to another. Therefore, the majority of patients included in this study are at risk of intramuscular administration, which will likely lead to more variable rates of absorption of the allergen extract and potentially increase the risk of systemic reactions, including anaphylaxis.

To the best of our knowledge, this is the first study to assess the STMD in patients receiving SCIT. Allergen immunotherapy has traditionally been injected subcutaneously. However, there are no published data comparing the safety or efficacy of immunotherapy injected intramuscularly versus subcutaneously. In fact, based on our data, we believe that a significant number of patients presently undergoing SCIT are receiving the injections intramuscularly. Currently, the AAAAI recommends that the skin be lifted in order to avoid intramuscular injections, and that SCIT be injected using a syringe with a needle length between 9.5 and 13 mm [6]. There has never been a study in immunotherapy patients to confirm that “pinching” or “lifting” the skin increases the STMD. However, a “two finger pinch” technique lead to an increase from 5.1 ± 1.8 mm to 9.0 ± 2.8 mm of the STMD in the arms and thighs of diabetic children in an open-label study [17]. In our clinical experience, the change in STMD with “pinching” the skin is quite variable from patient to patient. Also, the results of this current study suggests that the majority of adult patients (91%) have a STMD of >4 mm, with only 55% and 37% having depths of >8 mm and >10 mm, respectively (Table 2). Therefore, even if the SCIT were injected at a 45° angle with the *BD Safety Glide™ Allergy* needle inserted completely, the estimated depth of injection would be approximately 9.2 mm using the Pythagorean theorem. In our subjects, well over 50% of patients would have received the SCIT intramuscularly using this technique. Therefore, to reduce the risk of accidental intramuscular allergen administration, a standard needle length of 4 mm may be preferred in patients receiving SCIT.

As noted above, we could not identify any studies addressing the issues of STMD in the allergen immunotherapy literature. But there are a number of studies addressing similar issues in diabetics. In insulin-requiring diabetic patients, insulin therapy is usually injected or infused subcutaneously. Vaag et al. found higher serum insulin levels if insulin was injected intramuscularly versus subcutaneously [18]. As well, the blood flow and rates of insulin absorption may vary even at different subcutaneous sites in the abdomen [19,20]. Diabetic patients have differing STMD where insulin is injected. So insulin needles are available at various lengths including 5, 8 and 12.7 mm. A recent study showed that an even shorter 4 mm needle was associated with a reduced risk of intramuscular insulin injections compared to a 6 mm needle [21]. Another study revealed that 86% of diabetics received insulin intramuscularly with a 12.7 mm long needle [17]. An article has suggested and predicted that the use of needles shorter than 6 mm will be more widely accepted in diabetes with more evidence for their safety and efficacy [22]. This accommodation should help to ensure that the dosage of insulin is injected into the correct tissue compartment for each patient. Currently, it is not standard practice to perform ultrasounds on diabetic patients to ensure that the appropriate needle length is used.

**Figure 2:**
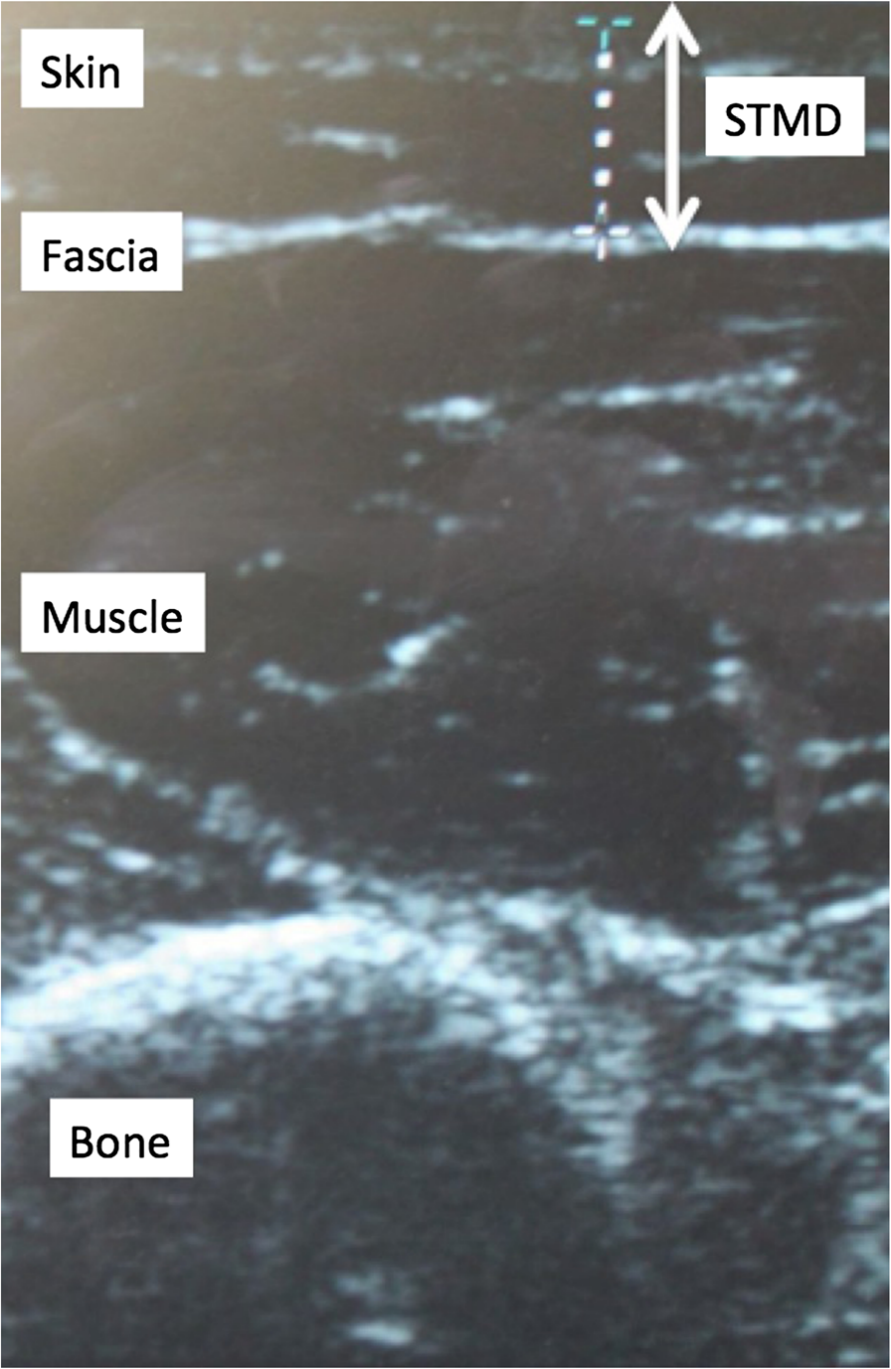
Image of ultrasound showing skin-to-muscle depth (STMD).

Based on the findings of this current study, we feel that some modifications to routine clinical practice may be warranted to reduce the risk of inadvertent intramuscular administration of allergen immunotherapy. Firstly, an ultrasound could be performed on all patients receiving SCIT in order to confirm their STMD. This approach would ensure that healthcare workers administering the injections are aware of the real depth to the intramuscular space. The potential drawbacks to the routine use of ultrasounds, however, are their associated costs and the expertise and time required to complete the procedures. Nonetheless, we feel that it would be straightforward for most allergists to learn how to read these relatively simple ultrasounds (see Figure 2). The cost of ultrasound machines has also decreased recently making this technology financially attainable in most medical clinics.

Secondly, injections could be administered to a depth of 4 mm or less using the allergy needles already available; this requires that the syringe not be pushed to maximal depth. According to the results of our study, this would decrease the risk of intramuscular injection to less than 9%. Nonetheless, injecting even to this depth may lead to intramuscular administration in patients with very low STMD (i.e., < 4 mm).

Lastly, the standard needle length of allergy syringes used for SCIT could be decreased to 4 mm. Again, even this needle length would still leave some patients at risk of intramuscular injections. However, if the needles were injected at a 45° angle to the skin, there would be less risk of intramuscular administration. Also, intradermal injections should be avoided.

There are some limitations to the present study that should be noted. First, the study was completed in one allergy clinic in Southwestern Ontario, Canada. All of the subjects lived in the same geographic area and most subjects were Caucasian. Different populations will likely have different BMIs and, therefore, different STMD. Secondly, although BMI correlated with STMD, it was not a perfect predictor of the STMD. In fact, everyone will have a different STMD, and the STMD may change over time in the same patient. Thirdly, we only assessed adults in this study. Children are more likely to have a lower STMD than adults and, hence, younger patients may be at even higher risk of inadvertent intramuscular injection during SCIT. As noted previously, “pinching” the skin may lead to an increase in the STMD. We did not measure STMD after “pinching”. Finally, only one physician performed and read all of the ultrasounds in an unblinded fashion. This may have lead to observer bias.

## Conclusion

Most patients receiving SCIT in our study have a STMD less than the needle length of a standard allergy syringe (13 mm). These patients are at risk of receiving injections intramuscularly, which may increase the risk of anaphylaxis. Using a syringe with a needle length of 4 mm given at a 45° angle to the skin would likely decrease this risk. A larger, multicenter study assessing the STMD of patients on SCIT is recommended in order to confirm the findings of this single-center study. Studies assessing the safety and efficacy of SCIT versus intramuscularly injected immunotherapy could also be considered, although the ethical considerations in completing trials of this nature may be prohibitive.

## Abbreviations

AAAAI: American academy of allergy, asthma and immunology; BMI: Body mass index; SCIT: Subcutaneous immunotherapy; STMD: Skin-to-muscle depth.

## Competing interests

The authors declare that they have no competing interests.

## Authors’ contributions

All authors contributed to the study design. LK, RP and CE were responsible for data collection. IN and AD performed statistical analyses. LK and IN drafted the manuscript. All authors revised the manuscript and approved the final version.
